# *Fusobacterium necrophorum* in necrotic laryngitis of feedlot cattle

**DOI:** 10.1093/jvimsj/aalag156

**Published:** 2026-08-03

**Authors:** Raghavendra G Amachawadi, Matt Miesner, Warren Beard, Laurie Beard, Daniel U Thomson, Tom Noffsinger, Kip Lukasiewicz, Harith M Salih, Haiyan Wang, Tiruvoor G Nagaraja

**Affiliations:** Department of Clinical Sciences, College of Veterinary Medicine, Kansas State University, Manhattan, KS 66506, United States; Department of Clinical Sciences, College of Veterinary Medicine, Kansas State University, Manhattan, KS 66506, United States; Department of Clinical Sciences, College of Veterinary Medicine, Kansas State University, Manhattan, KS 66506, United States; Department of Clinical Sciences, College of Veterinary Medicine, Kansas State University, Manhattan, KS 66506, United States; Production Animal Consultation, Manhattan, KS 66503, United States; Production Animal Consultation, Manhattan, KS 66503, United States; Production Animal Consultation, Manhattan, KS 66503, United States; Department of Clinical Sciences, College of Veterinary Medicine, Kansas State University, Manhattan, KS 66506, United States; Department of Statistics, College of Arts and Sciences, Kansas State University, Manhattan, KS 66506, United States; Department of Diagnostic Medicine/Pathobiology, College of Veterinary Medicine, Kansas State University, Manhattan, KS 66506, United States

**Keywords:** calf diphtheria, feedlot cattle, *Fusobacterium necrophorum*, necrotic laryngitis, tylosin resistance

## Abstract

**Background:**

Necrotic laryngitis is an important cause of respiratory distress and economic loss in feedlot cattle.

**Hypothesis/Objectives:**

Describe the clinical presentation, pathologic lesions, and microbiologic findings associated with necrotic laryngitis in feedlot steers.

**Animals:**

Four 20-month-old feedlot steers diagnosed with necrotic laryngitis. Clinically affected steers exhibited inspiratory stridor, increased respiratory effort, and flared nares, indicative of upper airway obstruction.

**Methods:**

Samples from inflamed nasopharyngeal tissues were collected endoscopically using guarded cytology swabs and submitted for culture, with concurrent blood collection for CBC and plasma and serum biochemistry analyses.

**Results:**

Endoscopic examinations identified laryngeal chondritis, mucopurulent diphtheritic membranes, and necrotic inflammation of the vocal folds. Aerobic cultures were negative, but anaerobic cultures yielded *Fusobacterium necrophorum* subsp. *necrophorum* and or *F. necrophorum* subsp. *funduliforme*. Hematologic evaluation indicated leukocytosis and increased plasma fibrinogen concentration, whereas serum biochemistry disclosed marked hyperglycemia and mild increases in hepatic enzyme activities, consistent with stress-related metabolic alterations. Antimicrobial susceptibility testing showed that *F. necrophorum* isolates were susceptible to β-lactams, tetracyclines, florfenicol, tiamulin, and trimethoprim/sulfamethoxazole, and resistant to aminoglycosides, fluoroquinolones, and with respect to macrolides, 4 strains were susceptible and 1 strain was resistant to tylosin, tilmicosin, and tulathromycin.

**Conclusions and clinical importance:**

These findings indicate involvement of either *F. necrophorum* subsp. *necrophorum* or subsp. *funduliforme* in necrotic laryngitis of feedlot cattle. Observed antimicrobial susceptibility patterns indicate ongoing susceptibility to β-lactams and tetracyclines, supporting their continued use as first-line therapeutic options.

## Introduction

Necrotic laryngitis, or calf diphtheria, caused by *Fusobacterium necrophorum,* is characteristically a disease of young cattle.^1^ The morbidity of feedlot cattle with necrotic laryngitis is typically 1%-2%, with cases reported in calves as young as 5 weeks and in cattle up to 2 years of age.^2^ The organism gains entry through mucosal damage in the larynx, often created by pre-existing upper respiratory infections involving viral and bacterial pathogens, such as infectious bovine rhinotracheitis virus and parainfluenza-3 virus, *Mycoplasma* spp., *Pasteurella* spp., and *Haemophilus* spp.^3^ In addition, physical trauma may create ulcerative lesions in the laryngeal mucosa, providing a portal for *F. necrophorum* to invade the submucosal tissues. *F. necrophorum, an anaerobe,* is the primary infectious agent associated with necrotic laryngitis in cattle, although other bacteria may be present.^3^ Once established, the organism causes necrosis of the laryngeal tissues, which may extend to the lower respiratory tract, leading to pneumonia and respiratory distress. Clinical signs include coughing, hypersalivation, dyspnea with open-mouth breathing, extended neck posture, and characteristic malodorous breath.^1^^,^^2^ If not identified and treated early, progressive laryngeal swelling and airway obstruction may necessitate emergency tracheostomy or surgical debridement. Antibiotic treatment, particularly with penicillin, is often effective in early cases. However, in chronic or advanced infections, the prognosis is guarded. In a retrospective cohort study, survival rate was 65.2%, with younger calves (<6 months) showing significantly higher mortality risk.^2^

Despite the well-documented role of *F. necrophorum* in necrotic laryngitis, no studies to date have identified which *F. necrophorum* subspecies, *necrophorum* or *funduliforme*, is involved. The 2 subspecies differ in colony and microscopic morphologies, growth characteristics, biochemical features, virulence potential, and hemagglutination activity,^4^ yet their relative involvement in laryngeal infections is not known. The virulence of *F. necrophorum* is attributed to several factors, most notably a high-molecular-weight leukotoxin that is cytotoxic to bovine neutrophils, lymphocytes, and macrophages, thus providing protection to the pathogen and compromising host immunity.^5^^,^^6^ In addition, outer membrane proteins (OMP) enhance the pathogen’s invasiveness by mediating adhesion to host epithelial cells.^7–9^ Recent studies have identified 4 high-affinity OMPs: OmpH (17.5 kDa), OmpA (22.7 kDa), CSP (66.3 kDa), and a novel OMPf (43 kDa), which play a role in host cell attachment, because combinations of antibodies strongly inhibited adhesions to host cells.^9^

Despite the known role of *F. necrophorum* in the pathogenesis of necrotic laryngitis, subspecies confirmation has not been reported. Determining the specific subspecies is essential to better understand disease pathogenesis and guide targeted therapeutic, prevention, and control strategies. Therefore, our primary objective was to identify the *F. necrophorum* subspecies involved in necrotic laryngitis in feedlot cattle. Secondary objectives included characterizing the antimicrobial susceptibility profiles of the isolates and describing the clinical and clinicopathologic findings.

## Materials and methods

### Clinical presentation

Four cases of approximately 20-month-old steers (ID tags 61, 63, 68, and 88) were presented to the Kansas State University College of Veterinary Medicine Veterinary Health Center with audible inspiratory stridor, increased respiratory effort, and flared nares. Mild bilateral mucoid nasal discharge was observed, as well as foul breath odor. Based on physical examination, upper airway inflammation was suspected, and endoscopy was performed to visualize the upper airway. An endoscope was passed through the nasal passage to the laryngeal area to examine the lesion.

### Sample collection

Samples of the diphtheritic membrane and discharge on the coronoid processes as well as the nasopharyngeal surface and septum were collected. A double-guarded mare brush cytology pipette was passed alongside the endoscope to obtain surface samples from the infected tissue under direct visualization. Samples were submitted to the laboratory for aerobic and anaerobic cultures. Additionally, blood was collected from the coccygeal vein for a complete blood count CBC and serum biochemistry.

### Complete blood count and serum chemistry

The CBCs were performed within 4 h using a Siemens ADVIA 2120i hematology analyzer (Siemens Healthcare, Erlangen, Germany) following the manufacturer’s protocol. Serum obtained after centrifugation was analyzed using the Roche Cobas c501 Clinical Chemistry Analyzer (Roche Diagnostics, Switzerland) according to the manufacturer’s procedures.

### Isolation, identification, and subspeciation of *F. necrophorum*

The samples were streaked onto blood agar plates and incubated anaerobically in a Glove Box. Presumptive *F. necrophorum* colonies were identified based on their characteristic morphology.^10^^,^^11^ Species-level identification was carried out using the RapID-ANA test kit (Thermo Fisher Scientific Inc.).^12^ Subspecies differentiation of *F. necrophorum* was performed based on sedimentation characteristics in broth and phosphatase activity.^13^ Isolates were further confirmed by PCR detection of the *heme* (*F. necrophorum*) and *gyrB* (*F. funduliforme*) genes.^13^^,^^14^

### Antimicrobial susceptibility testing

Minimum inhibitory concentrations (MICs) were determined using the broth microdilution method according to Clinical and Laboratory Standards Institute (CLSI) guidelines, with Sensititre™ Vet Bovine BOPO6F plates (Thermo Fisher Scientific), following the manufacturer’s instructions. The MIC values were interpreted and classified as susceptible, intermediate, or resistant based on CLSI breakpoints for the antimicrobials included in the BOPO6F Sensititre™ panel.

## Results

### Clinical and endoscopic findings

**Figure 1:**
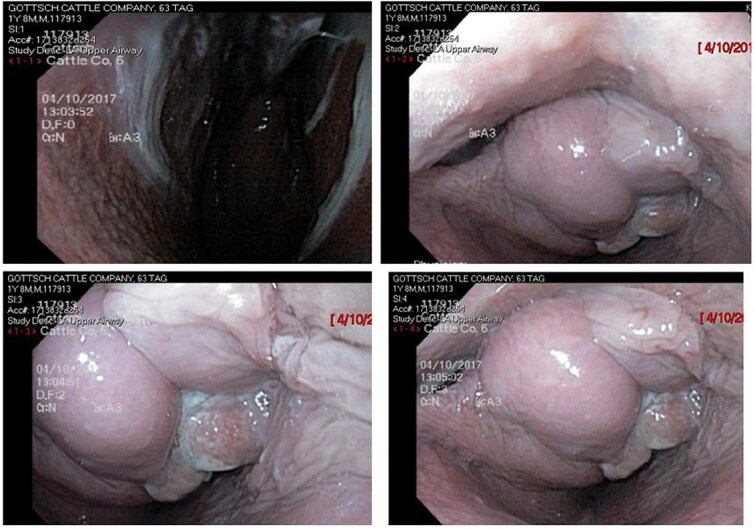
Endoscopic images of the larynx from steer 63 with necrotic laryngitis. The affected tissues exhibit marked inflammation, mucosal ulceration, and necrosis, particularly involving the arytenoid cartilages and surrounding structures.

Steers exhibited audible inspiratory stridor, increased respiratory effort, and flared nares, which became more pronounced after mild exercise. Physical examination identified mild to moderate increases in rectal temperature (102-103°F), heart rate (80-100 beats per minute), and respiratory rate (30-40 breaths per minute). All steers were alert and excitable, with one animal (ID 63) showing the most severe clinical signs. Constant inspiratory stridor and occasional expiratory stridor were present, with maximal noise localized over the laryngeal area. Palpation of the larynx induced coughing and increased respiratory effort. Mild bilateral mucoid nasal discharge and foul breath odor also were observed. Endoscopy identified nasopharyngeal lymphoid hyperplasia, laryngeal chondritis and paralysis, and a mucopurulent diphtheritic membrane on the vocal folds and coronoid processes, whereas the trachea and pulmonary airways appeared within normal limits (Figure 1).

### Bacterial culture and subspecies identification

Anaerobic bacterial cultures yielded *F. necrophorum* from all samples. Steer 88 was positive for subsp. *necrophorum*, steers 61, 63, and 68 were positive for subsp. *funduliforme*, and steer 61 was positive for both subspecies.

### Hematologic findings

Complete blood count data from all 4 steers are summarized in Table S1. Steers 61 and 68 exhibited leukocytosis, with neutrophilia. These findings reflect acute bacterial infection. Steers 63 and 88 had monocytosis, indicating a more chronic or resolving inflammatory phase. Lymphocyte counts were generally within or slightly above normal. Plasma fibrinogen concentrations were increased in 2 steers supporting an acute phase response. Plasma protein concentrations were slightly increased in 2 steers. Red blood cell indices, including erythrocyte counts, hemoglobin concentration, hematocrit, mean cell volume, and mean cell hemoglobin concentration, remained within normal ranges. Platelet counts were normal. The hematologic results indicated acute inflammatory state in 2 steers, whereas results in 2 steers were more consistent with a subacute or resolving condition.

### Serum biochemistry

Serum biochemistry profiles for the 4 calves are presented in Table S2. All animals were hyperglycemic, consistent with stress-induced hyperglycemia. Renal function was normal in all steers. Total protein, albumin, and calculated globulin concentrations were within reference limits. Serum electrolyte concentrations were normal. Liver enzymes activities included increased alkaline phosphatase activity in all calves and increased gamma-glutamyl transferase in 1 steer, indicating hepatobiliary stress. All serum samples were icteric, with 1 steer also showing mild hemolysis. Overall, the serum biochemistry findings were consistent with the CBC data, reinforcing the distinction between acute systemic involvement or resolving disease.

### Antimicrobial susceptibility results

The antimicrobial susceptibilities of the subsp. *necrophorum* (61A and 88A) and subsp. *funduliforme* isolates (61B, 63B, 68B) were evaluated against 18 antibiotics (Table 1). The subsp. *necrophorum* isolates were susceptible to β-lactams (ceftiofur, penicillin, ampicillin), tetracyclines (chlortetracycline, oxytetracycline), florfenicol, tiamulin, and trimethoprim/sulfamethoxazole, but exhibited resistance to aminoglycosides (gentamicin, neomycin), fluoroquinolones (danofloxacin, enrofloxacin), and macrolides (tylosin, tulathromycin, tilmicosin). A high MIC was also observed for sulphadimethoxine. In contrast, *F. funduliforme* isolates had more variable susceptibilities. All were susceptible to β-lactams, tetracyclines, florfenicol, and tiamulin, but uniformly resistant to aminoglycosides, fluoroquinolones, and spectinomycin. Four of the 5 strains, except subsp. *necrophorum*, strain 61A, were susceptible to the 3 macrolides tested. Susceptibility to trimethoprim/sulfamethoxazole was observed only in isolate 61B. Isolates 61B and 63B were also susceptible to the lincosamide, whereas the remaining isolates were not susceptible to this agent. Overall, both subspecies, *funduliforme* and *necrophorum,* were susceptible to first-line antimicrobial agents.

**Table 1:** Antimicrobial susceptibility patterns of *Fusobacterium necrophorum* subsp. *necrophorum* and subsp*. funduliforme* isolated from calves with necrotic laryngitis.

Antimicrobials	Strain 61A (Subsp. *necrophorum*)	Strain 61B (Subsp. *funduliforme*)	Strain 63B (Subsp. *funduliforme*)	Strain 68B (Subsp. *funduliforme*)	Strain 88A (Subsp. *necrophorum*)
Ampicillin	0.25 (S)	1 (S)	0.25 (S)	0.25 (S)	0.25 (S)
Ceftiofur	0.25 (S)	1 (S)	0.5 (S)	0.5 (S)	0.25 (S)
Chlortetracycline	8 (S)	8 (S)	8 (S)	4 (S)	4 (S)
Clindamycin	16 (R)	0.25 (S)	0.25 (S)	16 (S)	0.25 (S)
Danofloxacin	1 (R)	1 (R)	1 (R)	1 (R)	1 (R)
Enrofloxacin	2 (R)	2 (R)	2 (R)	2 (R)	2 (R)
Florfenicol	8 (S)	1 (S)	0.5 (S)	4 (S)	1 (S)
Gentamicin	16 (R)	16 (R)	16 (R)	16 (R)	16 (R)
Neomycin	32 (R)	32 (R)	32 (R)	32 (R)	32 (R)
Oxytetracycline	8 (S)	8 (S)	8 (S)	4 (S)	4 (S)
Penicillin	0.12 (S)	0.5 (S)	0.12 (S)	4 (S)	0.12 (S)
Spectinomycin	64 (R)	64 (R)	64 (R)	64 (R)	64 (R)
Sulphadimethoxine	256 (R)	256 (R)	256 (R)	256 (R)	256 (R)
Tiamulin	16 (S)	1 (S)	0.5 (S)	1 (S)	1 (S)
Tilmicosin	64 (R)	4 (S)	4 (S)	16 (S)	4 (S)
Tylosin	32 (R)	8 (S)	8 (S)	16 (S)	8 (S)
Trimethoprim/Sulfamethoxazole	2/38 (R)	2/38 (R)	2/38 (R)	2/38 (R)	2/38 (R)
Tulathromycin	64 (R)	8 (S)	8 (S)	16 (S)	4 (S)

Abbreviations: R = resistant; S = susceptible.

## Discussion

Our case series characterizes the clinical, endoscopic, bacteriologic, and hematologic findings in 4 feedlot steers with necrotic laryngitis, as well as the antimicrobial susceptibility patterns of the etiologic agents. It also characterizes the subspecies of *F. necrophorum* involved in necrotic laryngitis. *The subsp. funduliforme* was isolated in 3 of the 4 steers, whereas subsp. *necrophorum* was isolated from 2 steers. The clinical and endoscopic findings, laryngeal stridor, diphtheritic membranes, and chondritis are consistent with descriptions of classic necrotic laryngitis.^1^^,^^2^ The hematologic and biochemical changes reflect the expected consequences of severe, localized bacterial infection. Leukocytosis with neutrophilia in 2 steers indicated an acute inflammatory response, whereas slight monocytosis in 2 steers suggested a more chronic stage of disease or host immune response.^15–17^ The hyperglycemia is a well-documented stress response in critically ill cattle, and mild increases in hepatic enzyme activity likely resulted from systemic inflammation or hypoxia.^18^

The bacteriologic findings support the involvement of *F. necrophorum* as the primary bacterial species associated with the lesions, with both subsp. *necrophorum* and *funduliforme* identified, including a co-infection in one steer. Their known differences in virulence and leukotoxin production reinforce the pathogenic role of *Fusobacterium* in necrotic laryngitis.^19–21^ Only one of the 2 strains of subsp. *necrophorum* exhibited phenotypic resistance to tylosin, but the small sample size limits the ability to draw conclusions about tylosin resistance. We previously also found no significant differences in MIC values for *F. necrophorum* isolated from liver abscesses of cattle fed tylosin compared to those from cattle that did not receive tylosin, further supporting the notion that tylosin exposure does not consistently select for resistance in this species.^22^ Recently, another study reported tylosin resistance, based on genotypic and phenotypic analyses, in 2 strains of *F. necrophorum* isolated from liver abscesses.^23^ Collectively, the available evidence suggests that tylosin resistance in this organism remains uncommon, but because tylosin is widely used in feedlot cattle and commonly administered for necrotic laryngitis,^22^ continued surveillance is warranted to mitigate the potential risk of decreased clinical utility and treatment failure. The antimicrobial susceptibility and resistance profiles of the 5 strains are consistent with earlier reports describing antimicrobial susceptibility patterns in *F. necrophorum.*^24^^,^^25^ A previous study has identified macrolide resistance genes, such as *erm(B)*, in *F. necrophorum*,^26^ but the presence of such determinants was not evaluated in our current study. In contrast, susceptibilities to β-lactams and tetracyclines support their continued use as first-line antimicrobials.^1^ These findings emphasize the importance of antimicrobial susceptibility monitoring to guide effective treatment and mitigate resistance development in feedlot systems.

The hematologic and serum biochemistry results indicate that necrotic laryngitis elicits a measurable systemic response, characterized by neutrophilia in acute cases^15^^,^^16^ and monocytosis in animals with more chronic or resolving inflammation.^17^^,^^18^ Increased plasma protein and fibrinogen concentrations,^27^ hyperglycemia, and mild increases in liver enzyme and creatine kinase activities^28^^,^^29^ reflect systemic inflammation, stress, and secondary hepatic or muscle involvement. Despite these changes, erythrocyte indices, serum electrolyte concentration, and renal function test results remained normal, indicating preserved metabolic homeostasis even in advanced disease.^30^ The variation among steers, particularly the increased severity in one steer, emphasizes that necrotic laryngitis progresses and produces different systemic effects depending on disease stage and severity.^1^ Integrating CBC and serum biochemistry results with clinical assessment therefore can help veterinarians refine prognosis, identify calves with poor prognosis, and select more targeted interventions in feedlot practice.

Acute cases generally respond well to antimicrobials such as β-lactams and tetracyclines, whereas chronic cases may require surgical intervention with guarded outcomes. Antimicrobial susceptibility profiles identified the continued efficacy of β-lactams and tetracyclines as first-line treatments, but emphasize the need for careful selection of other drug classes because of emerging resistance.^3^ Integrating clinical evaluation, endoscopic assessment, hematology, and targeted microbiologic culture, including species-specific identification and susceptibility testing, is valuable for characterizing disease, informing research, and guiding the assessment of treatment protocols, thereby supporting evidence-based approaches and efforts to minimize antimicrobial resistance. Management practices that decrease risk factors may influence disease development, but we did not assess the impact of mucosal protection, nutrition, or environmental stress on necrotic laryngitis. Treatment failure has been reported in cases with disease progression involving deeper cartilaginous and supportive tissues of the larynx and pharynx,^2^ which is consistent with our observations of severe necrotic lesions in the cattle examined in our study. Infection begins with disruption of the mucosal barrier, initiating subsequent pathologic events.

## Conclusions

Necrotic laryngitis in feedlot cattle is associated with infection by *F. necrophorum*, and disease involvement may include either subsp. *necrophorum* or subsp. *funduliforme*. The observed antimicrobial susceptibility patterns indicate ongoing susceptibility to β-lactams and tetracyclines, supporting their continued use as first-line therapeutic options. These findings provide preliminary insights into pathogen characterization in feedlot systems and highlight the need for further studies with larger sample sizes to better inform clinical decision-making and antimicrobial stewardship.

## Supplementary Material

supplemental_tabs_1-2_aalag156

## Off-label antimicrobial declaration

Authors declare no off-label use of antimicrobials.

## Institutional animal care and use committee or other approval declaration

Authors declare no institutional animal care and use committee or other approval was needed.
