# Supplementary material for: Fusobacterium necrophorum in necrotic laryngitis of feedlot cattle
Source: J Vet Intern Med. 2026 Aug 3;40(4):aalag156. doi: 10.1093/jvimsj/aalag156 (PMC13430032; doi:10.1093/jvimsj/aalag156)
Supplement: supplemental_tabs_1-2_aalag156 [file supplemental_tabs_1-2_aalag156.docx]

**Supplementary Table 1.** Complete blood counts and plasma chemistry from steers clinically diagnosed with necrotic laryngitis.

| **Outcome** | **Steer 61** | **Steer 63** | **Steer 68** | **Steer 88** | **Reference Values** |
| --- | --- | --- | --- | --- | --- |
| Leukocyte count (K/µL) | 14.0 | 9.0 | 13.0 | 12.4 | 5.0 - 10.0 |
| Erythrocyte concentration (M/µL) | 7.77 | 9.97 | 8.02 | 8.87 | 6.0 - 12.00 |
| Hemoglobin (g/dL) | 12.7 | 14.4 | 13.6 | 13.9 | 8.0 - 15.0 |
| Cellular Hemoglobin (g/dL) | 12.6 | 14.3 | 13.6 | 13.7 | No Ref Interval |
| Hematocrit (calculated) (%) | 33.5 | 38 | 35.9 | 36.5 | 24 - 46 |
| Mean Cell Volume (fL) | 43.2 | 38.2 | 44.7 | 41.1 | 40.0 – 65.0 |
| Mean Cell Hemoglobin (pg) | 16.4 | 14.4 | 17.0 | 15.7 | 14.0 – 19.0 |
| Mean Cell Hemoglobin Concentration (g/dL) | 37.9 | 37.8 | 37.9 | 38.2 | 30.0 – 36.0 |
| RBC Distribution Width (%) | 21.9 | 20.5 | 18.4 | 20.0 | No Ref Interval |
| Platelet (electronic) (K/µL) | 522 | 517 | 301 | 317,000* | 100 – 800 |
| Segmented neutrophil concentration (K/µL) | 5.9 | 3.3 | 8.3 | 4.9 | 1.0 - 5.0 |
| Band neutrophil concentration (K/µL) | 0.0 | 0.0 | 0.0 | 0.0 | 0.0 - 0.2 |
| Lymphocyte Concentration (K/µL) | 7.4 | 4.7 | 3.6 | 5.8 | 2.5 - 7.5 |
| Monocyte concentration (K/µL) | 0.7 | 0.9 | 0.5 | 1.1 | 0.0 - 0.8 |
| Eosinophil concentration (K/µL) | 0.0 | 0.1 | 0.4 | 0.5 | 0.0 - 1.6 |
| Basophil concentration (K/µL) | 0.0 | 0.1 | 0.1 | 0.0 | 0.0 - 0.2 |
| Platelet estimate | Adequate | Adequate | Adequate | Adequate |  |
| Platelet features |  | Clumps | Clumps | Many clumps |  |
| Erythrocyte features | No polychromasia seen, moderate crenation | No polychromasia seen | None seen polychromasia, marked crenation | No polychromasia seen |  |
| Leukocyte features | None | Occasional reactive lymphocyte | None | Occasional reactive lymphocyte |  |
| Hematocrit (spun) (%) | 35 | 40 | 39 | 39 | 26 - 42 |
| Plasma protein by refractometry (g/dL) | 9.1 | 9.0 | 8.7 | 8.6 | 7.0 - 9.0 |
| Fibrinogen (heat ppt) (mg/dL) | 600 | 900 | 700 | 600 | 300 - 700 |
| Gross appearance of plasma | Normal | Normal | Normal | Normal | - |

**Supplementary Table 2.** Serum biochemistry from steers clinically diagnosed with necrotic laryngitis.

| **Outcome** | **Steer 61** | **Steer 63** | **Steer 68** | **Steer 88** | **Reference Values** |
| --- | --- | --- | --- | --- | --- |
| Glucose (mg/dL) | 91 | 125 | 103 | 84 | 29 - 73 |
| Urea nitrogen (mg/dL) | 8 | 11 | 7 | 8 | 9 - 24 |
| Creatinine (mg/dL) | 0.9 | 1.0 | 0.8 | 1.0 | 0.5 - 1.6 |
| Protein, total (g/dL) | 8.6 | 8.1 | 8.3 | 8.0 | 6.0 - 9.0 |
| Albumin (g/dL) | 3.7 | 3.7 | 3.4 | 3.3 | 3.1 - 4.3 |
| Globulin, calculated (g/dL) | 4.9 | 4.4 | 4.9 | 4.7 | No Ref Interval |
| Calcium, total (mg/dL) | 9.6 | 9.7 | 10.1 | 9.6 | 8.1 - 10.3 |
| Phosphorus (mg/dL) | 6.1 | 6.1 | 6.2 | 7.2 | 4.9 - 9.0 |
| Sodium (mmol/L) | 144 | 142 | 145 | 144 | 138 - 155 |
| Potassium (mmol/L) | 4.7 | 4.0 | 4.2 | 4.4 | 4.2 - 6.3 |
| Chloride (mmol/L) | 98 | 97 | 97 | 100 | 92 - 117 |
| Bicarbonate (mmol/L) | 28.1 | 27.6 | 31.2 | 26 | 21 - 31 |
| Anion gap, calculated (mmol/L) | 24 | 22 | 22 | 23 | No Ref Interval |
| Sodium Potassium Ratio (mmol/L) | 31 | 36 | 35 | 33 | No Ref Interval |
| Aspartate transaminase P5P (mmol/L) | 104 | 71 | 159 | 114 | 53 - 156 |
| Alkaline phosphatase (U/L) | 115 | 93 | 124 | 86 | 20 - 76 |
| Gamma-glutamyltransferase (U/L) | 30 | 55 | 30 | 12 | 10 - 39 |
| Sorbitol Dehydrogenase (U/L) | 13.4 | 11.0 | 18.5 | 17.7 | 6.1 - 18.4 |
| Creatine kinase (U/L) | 340 | 270 | 514 | 132 | 171 - 357 |
| Gross appearance of serum | Icterus | Icterus | Icterus | Mild hemolysis, icterus | - |
